# Factors associated with frequent high-cost individuals with cystic fibrosis and their healthcare utilization and cost patterns

**DOI:** 10.1038/s41598-023-35942-7

**Published:** 2023-06-01

**Authors:** Sameer Desai, Wei Zhang, Jason M. Sutherland, Joel Singer, Bradley S. Quon

**Affiliations:** 1 School of Population and Public Health, University of British Columbia, Vancouver, BC Canada; 2 Centre for Health Evaluation and Outcome Sciences, St. Paul’s Hospital, Vancouver, BC Canada; 3 Centre for Health Services and Policy Research, University of British Columbia, Vancouver, BC Canada; 4 Centre for Heart Lung Innovation, University of British Columbia, St. Paul’s Hospital, #166 - 1081 Burrard Street, Vancouver, BC V6Z 1Y6 Canada; 5 Division of Respiratory Medicine, Department of Medicine, University of British Columbia, Vancouver, BC Canada

**Keywords:** Health services, Health care

## Abstract

Cystic fibrosis (CF) is a progressive multi-organ disease with significant morbidity placing extensive demands on the healthcare system. Little is known about those individuals with CF who continually incur high costs over multiple years. Understanding their characteristics may help inform opportunities to improve management and care, and potentially reduce costs. The purpose of this study was to identify and understand the clinical and demographic attributes of frequent high-costing CF individuals and characterize their healthcare utilization and costs over time. A longitudinal study of retrospective data was completed in British Columbia, Canada by linking the Canadian CF Registry with provincial healthcare administrative databases for the period between 2009 and 2017. Multivariable Cox regression models were employed to identify baseline factors associated with becoming a frequent high-cost CF user (vs. not a frequent high-cost CF user) in the follow-up period. We found that severe lung impairment (Hazard Ratio [HR]: 3.71, 95% confidence interval [CI], 1.49–9.21), lung transplantation (HR: 4.23, 95% CI, 1.68–10.69), liver cirrhosis with portal hypertension (HR: 10.96, 95% CI: 3.85–31.20) and female sex (HR: 1.97, 95% CI: 1.13–3.44) were associated with becoming a frequent high-cost CF user. Fifty-nine (17% of cohort) frequent high-cost CF users accounted for more than one-third of the overall total healthcare costs, largely due to inpatient hospitalization and outpatient medication costs.

## Introduction

Healthcare resource use and costs associated with cystic fibrosis (CF) care have increased dramatically over the past two decades^1–5^. The cost of CF care can vary widely depending on a number of factors, including the health status of the CF population, practice patterns, availability of treatments (e.g., cystic fibrosis conductance transmembrane [CFTR] modulators) and the type of healthcare system (public vs. private) of each country. In Europe and Canada, recent cost estimates range from $20.0–$46.2 K CDN per individual^6–8^. However, in countries where CFTR modulators are more widely available, the cost estimates can be even higher^6,7^. In comparison, in the United States, where CFTR modulators are increasingly used, the cost of CF care is much higher^8^. Despite variations in cost estimates across different countries and regions, the trend of increasing costs over time for individuals with CF has been well-documented in the literature. Most of the increase in costs has been attributed to expensive specialty drugs such as CFTR modulators, dornase alfa, aztreonam, and other nebulized therapies^7–11^.

Measuring healthcare costs and understanding its main drivers provides valuable insights to health organizations, program planners and budget analysts to efficiently manage population budgets^12^. Recent studies have found that age, sex, lung function, genotype, chronic infection with *Pseudomonas aeruginosa* or *Burkholderia cepacia complex*, and CF-related diabetes (CFRD) increase healthcare costs^2,4,5,13^. However, most of these previous studies have identified associations between clinical and demographic factors and cost based on cross-sectional analyses. The use of longitudinal data can offer a more in-depth understanding of the factors that are associated with healthcare costs. By examining changes over time rather than just single time-points, researchers can identify cost-influencing factors that are likely to have a temporal relationship with healthcare costs. This provides stronger evidence for relationships between the factors and healthcare costs. Furthermore, it enables a thorough evaluation of the health expenditure categories that are relevant to understanding the costs of individuals with CF and how they have evolved over time.

Recently, there has been growing interest among the health services community to identify segments of a patient population who use a disproportionate amount of healthcare resources, also known as “super-users”^14–17^. Super-users are high-costing individuals who account for a significant portion of public spending on healthcare services, products and technologies. Research has shown that high-cost individuals are a small proportion of the population (< 10%) whose needs may not be properly met^16,18^ and some may receive unnecessary or ineffective care^15,17^. The purpose of this study is to identify and understand the clinical and demographic attributes of frequent high-cost CF individuals and characterize their healthcare utilization and costs over time.

## Methods

### Data sources

The Canadian Cystic Fibrosis Registry (CCFR) was used to identify individuals with CF in British Columbia (BC), a province of Canada whose population exceeds five million insured residents. The BC healthcare system provides universal, publicly funded health services to all residents with CF. Coverage includes visits to specialized care such as that provided by CF clinics, hospitalizations, and also for some CF medications without direct financial barriers. However, some costs are not covered such as medication equipment and travel expenses. CF care in BC is centralized with large pediatric and adult clinics in Vancouver which service the mainland of BC and smaller pediatric and adult clinics in Victoria which service Vancouver Island. Both Vancouver clinics provide outreach care to rural health regions via telehealth and satellite in-person clinics. The CCFR captures individuals with a confirmed diagnosis of CF based on current diagnostic guidelines. The CCFR contains detailed demographic and clinical data on individuals who have provided consent at one of 43 accredited CF centers across Canada^19^.

Multiple linkable administrative databases from the BC Ministry of Health were used for determining individual’s health care costs. The medical services plan (MSP) claims database was used to identify spending on outpatient physician services^20^. A BC-specific provincial discharge abstract database (DAD), including various Canadian Institutes Health Information (CIHI) value-added elements (such as case mix groups, and resource intensity weights) were used for estimation of hospitalization costs^21,22^. The PharmaNet database captures population-based medications dispensed from all hospital outpatient pharmacies and community-based pharmacies in BC^23^. The national ambulatory care reporting system (NACRS) was used to identify data regarding emergency department (ED) visits^24^. Together, these administrative databases provide individual-level information on the majority of costs relating to outpatient services, inpatient hospitalizations, medications, and ED visits of all residents of BC. Access to data provided by the Data Steward(s) is subject to approval, but can be requested for research projects through the Data Steward(s) or their designated service providers. All inferences, opinions and conclusions drawn in this study are those of the authors and do not reflect the opinions or policies of the Data Steward(s). The study was approved by the research ethics board (REB) at St. Paul’s Hospital, Vancouver, Canada (PHC-REB #H10-00,235). All methods were carried out in accordance with relevant guidelines and regulations. Informed consent was obtained from all included CF individuals and/or their legal guardians for collection of registry data and a waiver of consent was granted from REB for the current analysis.

### Study design and analytic cohort

A retrospective cohort of newly diagnosed and prevalent individuals with CF in BC between January 1, 2009, and December 31, 2017, were selected for inclusion in the study. Individuals were probabilistically linked with the administrative databases based on patient name, date of birth, and sex using the RecordLinkage package^25^ in R statistical software^26^. More details about the linkage process is provided in the supplementary document (Fig. S1). To improve sensitivity of the CF cohort, the probabilistically linked individuals were further restricted to include only those with at least one inpatient hospitalization or outpatient physician claim with a diagnosis of CF using the ICD-9 or ICD-10 codes indicating CF (277.0 or E84.X). All resulting individuals formed the analytic cohort and were followed from study entry (i.e., year 2009) or year of first resource use (referred to as “index year”) if they had no observation in 2009 until the year of last resource use or death during the study period. Only individuals 6 years and older were included to ensure reliability and consistency in clinical measurements.

### Definition of frequent high-cost CF users

Many studies examining high-cost patients rely on episodic, short-term healthcare costs from a single time-point to define super-users rather than those who are high-cost users in multiple years. In this study, an individual was categorized as a “frequent high-cost user” if their annual total healthcare cost was in the top 10% (top decile) of costs for two consecutive years or 50% of their follow-up time if it was more than two years from 2009 to 2017. Thus, individuals with follow-up time less than two years were excluded from our study. Individuals not falling into this category were classified as “not frequent high-cost” CF users.

**Figure 1:**
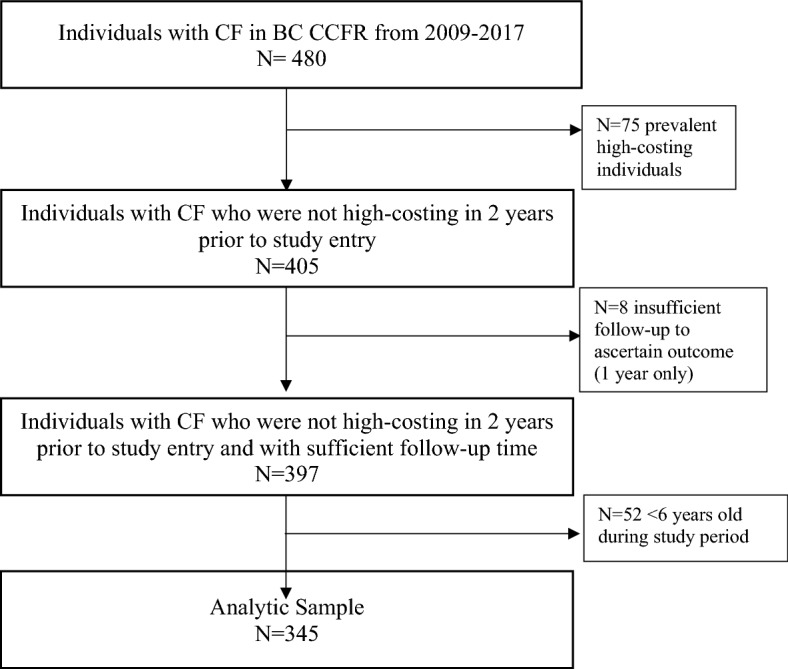
Flow diagram for inclusion of people with CF in study cohort.

Each individual’s total annual healthcare costs associated with outpatient services, inpatient hospitalizations, outpatient medications, and ED services were summed and adjusted for inflation using the consumer price index for health and personal care in BC^27^. As such, direct costs were assessed from the perspective of the public payer, the BC Ministry of Health. To focus on the factors that may lead to becoming a new (incident) frequent high-cost user during the study period, we excluded individuals who were already high-costing in the two years prior (2007 and 2008) to the index year. Figure 1 shows the flowchart for the creation of the analytic cohort.

### Factors of interest

*Patient demographic* variables such as age category at index year (6–11, 12–18, 19–40, 40+), sex, and region of residence according to Health Authority (Fraser, Vancouver Coastal, Interior, Vancouver Island, Northern) were assessed. The Health Authorities in BC are organized based on regional boundaries. The Vancouver Island health authority primarily serves individuals living on Vancouver Island, while the Vancouver Coastal health authority serves the metro Vancouver area. The Fraser Health authority is adjacent to Vancouver Coastal but extends eastward, while the Northern and Interior regions are remote, largely rural areas covering the northern and southern interior parts of BC, respectively. *Clinical characteristics* were also evaluated and summarized. Table 1 defines each factor of interest in more detail. Factors of interest for both groups were evaluated in the index year of the follow-up period.

**Table 1:** Definitions of factors of interest. All factors evaluated at baseline (defined as within 2 years of index year).

Factor of interest	Definition	Prior literature
BMI (body mass index)	Classified as underweight (≤ 12% for children and < 18.5 kg/m^2^ for adults), adequate weight (13–84% for children and 18.5–24.9 kg/m^2^ for adults), or overweight (> 84% for children, ≥ 25 kg/m^2^ for adults) using percentile and absolute value methods for children and adults^28,29^	Johnson et al., Ouyang et al.: Higher BMI associated with increased ln costs Gu et al., MIcoch et al.: Malnutrition/underweight not associated with increased costs
*Burkhholderia Cepacia Complex (BCC)*	Positive cultures for BCC = Yes, otherwise = No	Johnson et al., Gu et al., : BCC + increased ln costs MICoch et al.: Chronic BCC + not associated with increased ln costs
CF-related diabetes (CFRD) requiring treatment	Patient regularly prescribed any glucose lowering therapy (oral agents, insulin, etc.) and/or CFRD considered permanent condition (min. 3 months of therapy) = Yes, otherwise = No -Number of years since CFRD diagnosis date	Sawicki et al., Ouyang et al.: Increased costs for individuals with CFRD
CF transmembrane conductance regulator (CFTR) mutation type	-F508del homozygous, F508del heterozygous, two non-F508del, one mutation (any) present only, or unknown Presence of F508del = Yes, otherwise = No	Gu et al.: Homo F508del relative to heterozygous for F508del or patients presenting any other mutations of the CFTR gene had higher costs MICoch et al., Sawicki et al.: Mutation type was not associated with increased ln costs
Lung function (ppFEV_1_)	Individual’s forced expiratory volume in 1 s (FEV_1_), from the first “stable” visit of the year (i.e., patient not being treated for pulmonary exacerbation)^30^ and expressed as a percentage of predicted values (ppFEV_1_)^31^ and classified into categories (normal [≥ 90% ppFEV_1_], mild [70–89%], moderate [40–69%] or severe [< 40%]) corresponding to different levels of lung function impairment^30,32,33^	Johnson et al., Gu et al., MICoch et al., Van Gool et al., and Ouyang et al.: Higher lung function associated with decreased costs
Lung transplantation status	No transplant, peri-transplant phase (within two years pre- or post- transplant), and > 2 years post-transplant)	Van Gool et al., Ouyang et al
Physician-diagnosed CF-related complications	Sum of all available complications (distal intestinal obstructive syndrome, liver cirrhosis/portal hypertension, massive hemoptysis, nasal polyps/sinusitis, pneumothorax) Presence of each complication = Yes, otherwise = No	Ouyang et al.*:* Sinusitis associated with higher costs
*Pseudomonas aeruginosa* (*PA*)	Positive cultures for *PA* = Yes, otherwise = No	Johnson et al., Gu et al., MIcoch et al., Sawicki et al., Ouyang et al.: *PA* + Increased ln costs
Psychiatric medication use (proxy for mental health disorder)	Use of any psychiatric medication = Yes, otherwise = No	None found

All evaluated demographic and clinical factors were obtained from the CCFR with the exception of region of residence (obtained from MSP) and psychiatric medication use (PNET). Pancreatic insufficiency is collected as a time-invariant variable in the CCFR (no associated dates about the diagnosis) and therefore it was not evaluated as a potential factor as part of the multivariable analysis because we could not ascertain when an individual became pancreatic insufficient relative to becoming frequently high-cost.

### Characterizing utilization and costs

Healthcare utilization and direct costs were categorized into outpatient services, inpatient hospitalizations, outpatient medications, and ED services and examined over time from 2009 to 2017.

### Statistical analysis

Patient demographics and clinical characteristics were described using simple descriptive statistics (e.g., means, medians, and proportions).

To identify factors associated with becoming frequent high-cost users (Yes vs. No) over the study period, we first modelled each potential factor individually (univariable analysis) in a Cox regression model. The follow-up time was measured as the duration (in years) between the index year to being classified a frequently high-cost CF user based on the definition above. Individuals who did not become frequent high-cost users were censored at their last year of resource use. To construct our final multivariable model, we created a “base” model that included clinically important variables that are known to be associated with high costs (e.g., ppFEV_1_, *PA* positivity, transplant) based on the literature^3,5,34,35^ and demographic variables (age and sex). All other variables were added separately to the base model and those that were found to have *P*-values below the a priori defined cutoff (*P* < 0.25) were retained for inclusion in the final model. We chose this threshold to avoid the possibility of missing potentially important variables, which has been a common practice in previous studies^36–40^. The final model was selected by backward elimination using Akaike model criterion (AIC)^41^.

We also ran separate regression models for children (6–18) and adult (19+) age groups to reflect inherent differences in disease severity and cost of care. Variance inflation factors were used to detect multi-collinearity among the final factors. It provides an index that measures how much of the variance of a resulting regression co-efficient is increased because of multi-collinearity. Multi-collinearity occurs when two or more factors are highly correlated with each other, making it difficult to determine the unique contribution of each factor to the model. A variance inflation factor of greater than 1 indicates increasing levels of multi-collinearity^42^.

The level of significance was set at *P* < 0.05 for the final model and all reported *P*-values reflect two-tailed tests. Results from the models were presented as hazard ratios (HRs) with their corresponding 95% confidence intervals (CIs). Any missing data in the index year was replaced by the previous year’s data, if available (this evaluation period referred to as “baseline” hereafter). BMI and ppFEV_1_ still had some missing data (~ 12%) and multiple imputation using the fully conditional specification algorithm^43^ was used to account for this missing data. The proportional hazards assumption was tested (and satisfied) for each variable using Schoenfeld residuals^44^.

To characterize the sources of healthcare costs over time, we categorized annual costs of both cost groups by outpatient services, inpatient hospitalizations, outpatient medications, and ED services and calculated its percentage of total annual costs for each calendar year.

### Sensitivity analyses

Various sensitivity analyses examined the robustness of the main results. It was inevitable that any individual using CFTR modulators would be categorized as frequently high-cost after two years of use because of their high annual cost^45^ (commercial list price of $300 K per year per individual). Moreover, their continued use could impact subsequent annual costs as it improves patient outcomes and may reduce healthcare utilization^46–48^. As such, we conducted two sensitivity analyses exploring the effects of CFTR modulators on our overall results. The first sensitivity analysis excluded CFTR modulator costs from the total costs prior to classification as a frequent high-cost user or not, and the second analysis censored observations at the time of CFTR modulator initiation. We also re-ran the models employing different definitions of the outcome. We first only included individuals as “frequent high-cost” who had their costs in the top decile for 50% of their follow-up time only (i.e., do not classify individuals as “frequent high-cost” who were in the top decile for two consecutive years but less than 50% of their follow-up time). Second, instead of requiring two consecutive years to be classified as “frequently high-costing,” three consecutive years was also examined.

## Results

In total, 345 individuals were in the final analytic sample from 2009 to 2017 with a median follow-up time of 9.0 years (range: 2–9 years). There were 22 lung transplanted individuals and 20 deaths over 2596 person-years (7.7 deaths per 1000 person-years) from 2009 to 2017. Fifty-nine individuals (17%) were considered frequent high-cost users during the study period, of which 39 (66%) were adults. Frequent high-cost users were more likely to be female, pancreatic insufficient, positive for *P. aeruginosa,* and have CFRD at baseline (Table 2). Almost 70% of the frequent high-cost group had moderate or severe lung impairment at baseline (vs. 33% in the not frequent high-cost group) and 11 (19%) died by the end of the study period (vs. 3% in the not frequent high-cost group). Other clinical characteristics at baseline are shown in Table 2*.*

**Table 2:** Baseline clinical characteristics for individuals with CF in BC.

Characteristic	Total sample (N = 345)	Not frequent high-cost (N = 286)	Frequent high-cost (N = 59)
Age at diagnosis, y, median (IQR)	1 (0–8)	2 (0–9)	0 (0–4)
Age in baseline year, n (%)			
6–11	75 (22)	63 (22)	12 (20)
12–18	60 (17)	52 (18)	8 (14)
19+	210 (61)	171 (60)	39 (66)
Sex, n (%)			
Female	146 (42)	117 (41)	29 (49)
Male	199 (58)	169 (59)	30 (51)
Race, n (%)			
Caucasian	333 (97)	274 (96)	59 (100)
Non-Caucasian or unknown	12 (2.3)	≥ 5 (≥ 1.7)	< 5 (< 8.5)
Primary area of residence, n (%)			
Fraser	126 (37)	104 (36)	22 (37)
Interior	60 (17)	47 (16)	13 (22)
Northern	18 (5.2)	13 (4.5)	5 (8.5)
Island	59 (17)	50 (17)	9 (15)
Vancouver Coastal	82 (24)	72 (25)	10 (17)
Presence of F508del mutation, n (%)	305 (88)	250 (87)	55 (93)
CFRD on treatment, n (%)	68 (20)	52 (18)	16 (27)
CFRD years^a^, median (IQR)	4 (2–10)	4 (2–9.3)	6 (2–10.5)
Exocrine Pancreatic Insufficiency, n (%)	283 (82)	227 (79)	56 (95)
*P. aeruginosa*, n (%)	178 (52)	137 (48)	41 (69)
*B. cepacia complex,* n (%)	41 (12)	30 (10)	11 (19)
Underweight, n (%)	34 (11)	26 (11)	8 (15)
ppFEV_1_ category			
Normal	108 (36)	99 (40)	9 (17)
Mild	77 (25)	69 (28)	8 (15)
Moderate	82 (27)	62 (25)	20 (38)
Severe	35 (12)	19 (8)	16 (30)
Psychiatric medication use, n (%)	23 (6.7)	15 (5.2)	8 (14)
DIOS, n (%)	30 (8.7)	22 (7.7)	8 (14)
Liver cirrhosis, n (%)	7 (2)	< 5 (< 1.7)	≥ 5 (≥ 8.5)
Massive hemoptysis, n (%)	< 5 (< 1.4)	< 5 (< 1.7)	< 5 (< 8.5)
Pneumothorax, n (%)	< 5 (< 1.4)	< 5 (< 1.7)	< 5 (< 8.5)
Nasal polyps, n (%)	78 (23)	63 (22)	15 (25)
No. of complications, n (%)	0 (0–1)		
No complications	231 (67)	198 (69)	33 (56)
One complication	86 (25)	69 (24)	17 (29)
Two complications	25 (7.2)	17 (5.9)	8 (14)
Three complications	< 5 (< 1.4)	< 5 (< 1.7)	< 5 (< 8.5)
Transplantation status, n (%)			
No transplant	322 (93)	271 (95)	51 (86)
Peri-transplant	8 (2.3)	< 5 (< 1.7)	≥ 5 (≥ 8.5)
≥ 2 years post-transplant	15 (4.3)	≥ 5 (≥ 1.7)	< 5 (< 8.5)
CFTR modulator use in follow-up, n (%)	46 (13.3)	27 (9.4)	19 (32)
Lung transplantation in follow-up, n (%)	22 (6.4)	< 5 (< 1.7)	≥ 5 (≥ 8.5)
Death, n (%)	20 (5.8)	9 (3.1)	11 (19)
Follow-up time, y, median (IQR)	9.0 (9–11)	9 (7–9)	9 (5.5–9)
Follow-up time, y, mean (SD)	7.5 (2.3)	7.6 (2.2)	7.1 (2.5)

*y* years, *IQR* interquartile range, *SD* standard deviation, *CFRD* CF-related diabetes.

^a^Only applicable to those who have CFRD.

Cells containing less than 5 counts are suppressed and denoted as < 5; when counts less than 5 can be estimated with information from other categories, the smallest next category was replaced with “ ≥ 5 (≥ %)”.

### Factors associated with frequent high-cost users

On univariable analysis, lower lung function (moderate and severe) at baseline was associated with becoming a frequent high-cost user in the follow-up period (Table 3). The peri-transplant phase (i.e., 2 years before or 2 years after transplant) and increasing number of complications were associated with frequent high-cost users. Specifically, having liver cirrhosis at baseline was associated with becoming a frequent high-cost user in the study period.

**Table 3:** Results from cox regression model to identify variables associated with persistent high-cost users * *P* < 0.25, **P < 0.05.

Factors	Univariable HRs (95% CIs)	Final Multivariable HRs (95% CIs)
Patient demographics		
Female Sex (ref: Male)	1.28 (0.77–2.13)	1.97 (1.13–3.44)**
Health region (ref: Vancouver Coastal)		
Fraser	1.31 (0.62–2.77)	
Vancouver Island	1.28 (0.52–3.16)	
Interior	1.72 (0.75–3.92)*	
Northern	2.46 (0.84–7.18)*	
Age (ref: 6–11 yrs)		
12–18	0.64 (0.26–1.58)	
19–40	1.19 (0.62–2.30)	
40+	0.43 (0.15–1.21)*	
Clinical characteristics		
Presence of F508del mutation (ref: No)	1.92 (0.70–5.29)*	
CFRD (ref: No)	1.56 (0.88–2.78)*	
No. years since CFRD diagnosis (per 1-year since diagnosis)	1.04 (0.99–1.09)*	
*P. aeruginosa* (+ ve)	1.69 (0.99–2.88)*	1.14 (0.64–2.05)
*B. cepacia complex* (+ ve)	1.63 (0.82–3.21)*	
Underweight BMI (ref: not underweight)	1.51 (0.71–3.19)	
ppFEV_1_ category (ref: Normal)		
Mild	1.16 (0.45–3.00)	0.91 (0.36–2.34)
Moderate	2.95 (1.34–6.47)**	2.44 (1.13–5.25)**
Severe	7.05 (3.11–15.97)**	3.71 (1.49–9.21)**
Lung transplanted (ref: No)		
Peri-transplant phase	5.60 (2.54–12.36)**	4.23 (1.68–10.69)**
≥ 2 year post-transplant	0.77 (0.11–5.58)	0.87 (0.10–7.41)
No. of complications (per 1-complication increase)	1.70 (1.19–2.43)**	
Psychiatric medication use (ref: None)	2.01 (0.95–4.23)*	1.65 (0.76–3.60)
DIOS (ref: No)	1.43 (0.68–3.01)	
Liver cirrhosis (ref: No)	10.99 (4.33–27.90)**	10.96 (3.85–31.20)**
Hemoptysis (ref: No)	2.17 (0.53–8.91)*	
Nasal polyps (ref: No)	1.17 (0.65–2.11)	
Pneumothorax (ref: No)	1.53 (0.21–11.07)	

On multivariable analysis, age was found to have a variance inflation factor (VIF) > 2, indicating collinearity with other factors and therefore was removed from the final model. Female sex (HR: 1.97, 95% CI: 1.13–3.44), peri-transplant phase (HR: 4.23, 95% CI: 1.68–10.69), moderate lung function impairment (HR: 2.44, 95% CI: 1.13–5.25), severe lung function impairment (HR: 3.71, 95% CI: 1.49–9.21), and liver cirrhosis (HR: 10.96, 95% CI: 3.85–31.20) were all associated with frequent high-cost users.

Exclusion of CFTR modulator costs from total costs or censoring observations post-CFTR modulator initiation did not alter the results (Table S1). Similarly, retainment of age category in the final model also did not change the associations (Table S2). When the final model was re-run on different definitions of the outcome, all point estimates remained in the same direction but some became less precise due to a smaller number of individuals meeting the definition (Table S3).

When the regression model was run separately on children (6–18 years of age), having complications was the only factor associated with frequent high-cost users (HR: 7.86, 95% CI: 2.10–29.30) (Table S4). Relationships between transplant status, CFRD, psychiatric medication use, individual complications and frequently high-costing users were not estimated because none or very few children (< 5) had these conditions. The final results did not change when the analysis was restricted to adults (Table S5).

### Healthcare resource utilization and cost patterns of frequent high-cost users

Frequent high-cost users represented 59 individuals (17%) of the cohort and accounted for $2.1 M and up to $5.8 M (32–45% of the overall total healthcare costs) in any given year over the study period (Fig. S2).

**Figure 2:**
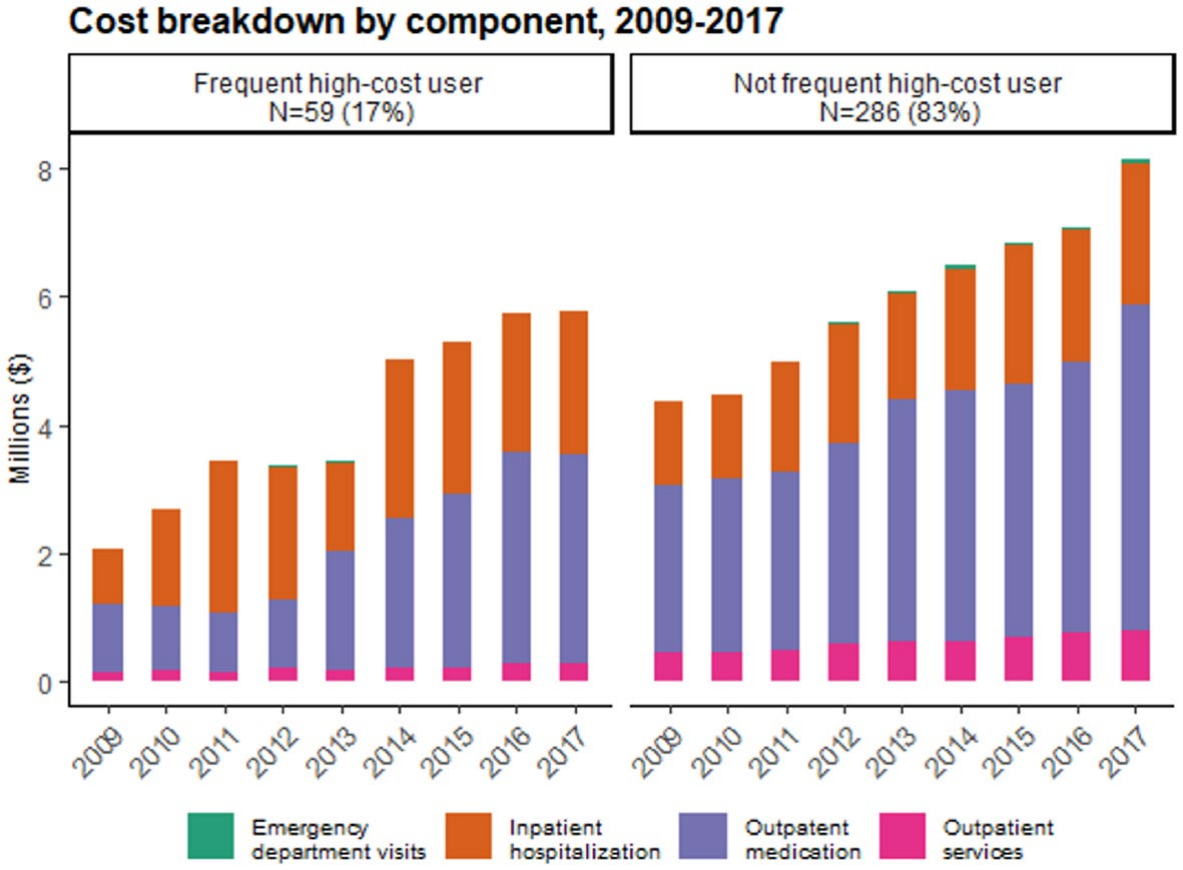
Cost breakdown by broad service categories for each cost, 2009–2017.

Median annual (per-patient) total costs for frequent high-cost users increased from $41 K in 2009 to $103 K in 2017 (Fig. S3). Frequent high-cost users had more specialist visits, outpatient medication claims, inpatient hospitalizations, and emergency department visits (Table S8) than not frequent high-cost users (Table S9) during the follow-up period. The main source of healthcare costs for frequent high-cost users was initially inpatient hospitalizations, accounting for over 50% of overall total direct healthcare costs. Since 2013, spending on outpatient medications has consistently been the main component of healthcare costs. Outpatient medications were the main source of costs for not frequent high-cost users throughout the study period (Fig. 2). Similar patterns were observed when examining each cost component as a proportion of overall total costs (Fig. S4).

When outpatient medications were broken down further, dornase alfa and digestive enzymes contributed the most to outpatient medication costs for both cost groups from 2007 to 2012 (Fig. S5). In 2013, 5 individuals initiated CFTR modulators and by 2017, this number increased to 46 individuals. Over this time span, CFTR modulators represented the largest share of outpatient medication costs for frequent high-cost users. When CFTR modulator costs were excluded, the top 5 outpatient medications with the largest costs for frequent high-cost users in 2017 were dornase alfa (28%), digestive enzymes (19%), nebulized aztreonam (19%), nebulized tobramycin (15%), and “other” drugs (12%). These medications represent the majority of overall medication costs for both groups, however, there were differences in the utilization of these medications by frequent high-cost users compared to not frequent high-cost users (Fig. S6 and Tables S8–10).

## Discussion

Frequent high-cost users represented 17% of the cohort but accounted for a disproportionate share of costs accounting for one-third and in more recent years nearly one-half of the overall total healthcare costs. Moderate-to-severe lung impairment, peri-transplantation phase, liver cirrhosis, and female sex were the main factors independently associated with becoming a frequent high-cost user. The majority of the costs for frequently high-cost users involved inpatient hospitalizations but in more recent years, CFTR modulators have become the main driver of overall costs.

All prior studies in CF evaluating clinical factors associated with healthcare costs have found that lower lung function is associated with higher healthcare costs^2,5,35,49–53^. Costs for those with advanced lung disease escalate because they experience more complications, have more frequent hospitalizations, and are prescribed more medications and maintenance therapies to alleviate symptoms and prevent pulmonary exacerbations^54^. Relatedly, people with CF who were 2 years pre- or post-transplantation were more likely to be frequent high-cost users. Van Gool et al.^3^ and Ouyang et al*.*^1^ reported similar findings with transplanted patients in their cohorts. Our study also found that females were two times more likely to become frequent high-cost users than males consistent with previous studies^4,10,34^. It is well-known that females with CF have faster disease progression leading to worse outcomes and reduced survival^55–57^. Prior studies have speculated that increased healthcare utilization by females might be related to increased hospitalizations and more intensive treatments and this was confirmed in our study (Fig. S7)^4^.

Liver cirrhosis/portal hypertension was also found to be associated with frequent high-cost users. Developing liver cirrhosis, particularly with portal hypertension represents a severe complication in CF^57,58^. The majority of healthcare costs for these individuals were due to inpatient hospitalizations related to pulmonary exacerbations. There is a lack of proven therapies to prevent or treat liver cirrhosis/portal hypertension among individuals with CF^59^. Liver transplant is a viable option to improve outcomes^60^ but it is often performed too late in CF as severity tends to be underestimated relative to other causes of end-stage liver disease. Recent studies have demonstrated that lung disease deteriorates prior to liver transplantation which lead to increased hospitalizations and healthcare costs^61^. As such, this group of high-cost users might be considered for liver transplantation earlier to reduce healthcare costs.

In recent years, the cost of CFTR modulators has emerged as the single most significant expense for frequent high-cost users. These costs related to modulators may be even higher as the PharmaNet database will not include costs for individuals who received the drug through Vertex’s compassionate access program or clinical trials at no charge^11^. This is not surprising as the commercial list price of CFTR modulators per-patient is approximately $300 K but the exact price negotiated with both public and private payers is not publicly available^62^. However, multiple peer-reviewed publications and health technology assessments suggest that the average listed market price for these drugs is approximately $300 K^62–66^. When Ivacaftor (Kalydeco®) was first introduced in Canada in 2013, public coverage was limited to select individuals with gating mutations (e.g. G551D, R117H) on an exceptional case-by-case basis through the BC government and the sickest individuals (based on ppFEV_1_) were prioritized. Therefore, some CFTR modulators users were also frequent high-cost users with or without including CFTR modulator costs. Highly-effective CFTR modulators such as Elexacaftor/Tezacaftor/Ivacaftor (Trikafta®) and Ivacaftor (Kalydeco^®^) are the most effective treatment options for eligible individuals with CF and they have the potential to prevent moderate-to-severe lung disease if initiated earlier in life^67,68^. Recent data also demonstrates that clinical improvements can even be achieved for those with advanced lung disease following the introduction of Elexacaftor/Tezacaftor/Ivacaftor with a two-fold decrease in the need for lung transplantation^48,69^. Based on our study’s findings, moderate-to-severe lung function impairment and transplantation are strongly linked to frequent high-cost users, therefore, the prevention of these conditions with more wide-spread use of highly-effective CFTR modulators will reduce healthcare costs. Moving forward, the costs of CF care are expected to be primarily driven by CFTR modulators, as eligibility criteria become less stringent and more countries provide public reimbursement for these medications^10,11,66^. Some modulators are now covered for children as young as 4 months old^70^, and there is growing motivation to initiate these treatments as early as possible to prevent co-morbidities and complications and achieve the greatest long-term benefits which will lead to further shifts from inpatient to outpatient care^71^. However, the high costs of the CFTR modulators themselves must also be reduced to achieve any healthcare cost savings^62^.

Interestingly, approximately 30% of the frequent high-cost users in our cohort were either children (12 [60%], 6–11 years; 8 [40%], 12–18 years) or individuals with normal-to-mild lung disease based on lung function. Therefore, two exploratory post-hoc analyses were performed to characterize these sub-groups of interest further. Children who were frequent high-cost users were more likely to be female, positive for *P. aeruginosa* and had more complications (Table S6). Individuals from the normal-to-mild lung disease group who were high cost users were less likely to have a F508del mutation, were positive for *P. aeruginosa*, and had 1 or more complications (Table S7). Future confirmatory investigations with larger sample sizes for these sub-groups are necessary.

This study has several limitations that should be acknowledged. Firstly, as an exploratory study examining correlations over time, caution should be exercised in inferring causality from our data. However, we attempted to establish temporality in the relationship by including individuals who were at least 2 years free of being high-cost prior to cohort entry and measuring each factor prior to becoming a frequent high-cost user. It is important to note that we were unable to determine the exact timing of the development of certain factors, such as severe lung disease or liver cirrhosis at baseline, due to the lack of earlier data (i.e., “left truncation”). Including such prevalent factors can bias estimates due to various selection mechanisms^72,73^. If earlier information was available, calculating time since first diagnosis/exposure to study entry could allow a better understanding of how the duration of each prevalent factor at assessment impacts the outcome. For example, does the likelihood of becoming a high cost user differ between individuals with a shorter duration of severe lung disease (i.e., recently diagnosed) compared to those who have had it for a longer period of time? Furthermore, structural social determinants of health (e.g., education, income) and environmental exposures (e.g., indoor/outdoor air quality) could not be accounted for and may impact the development of some of the factors of interest. Additionally, the home and community care databases were not used in the present study and as such some costs were not be captured. Home and community care services, including palliative and long-term care, will be important services to monitor as individuals with CF continue to live longer lives. Future studies should attempt to include these important factors if available to better understand the complex interplay between factors of interest and cost groups. Also, some individuals (n = 20) died in the follow-up period and typically, health care costs are heavily skewed towards the end of life, with a significant portion of costs incurring closer to death^74^. Further analyses of these individuals were beyond the scope of the present study, especially with a small sample size. Understanding the healthcare utilization and costs of aging individuals with CF is crucial, and should be a priority for future health services research as more people with CF are living into their later years. Lastly, our study required a minimum of two years of data to accurately measure the frequent healthcare costs, which may have resulted in the exclusion of sicker patients, potentially biasing estimates and reducing generalizability. However, the eight patients who were excluded due to this criterion were not necessarily sicker; rather, they had episodic costs such as G-tube placement. Unfortunately, we were unable to determine if these costs were short-term or ongoing due to the lack of data beyond the first year. Generalizability also was not impacted (Table S12).

From a policy and practice perspective, it is recommended that CF care teams should closely monitor individuals with liver cirrhosis or those at high risk of developing it. By doing so, their condition can be prevented from deteriorating further and medical treatment or liver transplantation can be initiated earlier, reducing the need for future hospitalizations and result in cost savings for the healthcare system. However, to further understand and validate this novel finding, it is important to prioritize conducting more research into this area before recommending other cost reduction or quality improvement interventions. Second, it has been observed previously, and in our study, that higher healthcare costs are linked to individuals with greater health issues and higher healthcare needs. To mitigate future healthcare expenses, starting treatment with CFTR modulators at an earlier age may prove to be a viable strategy. By preventing or slowing down lung function decline earlier on in childhood, the long-term cost impact on healthcare systems can be reduced by having more healthier adults in the future—higher lung function, fewer transplants, and less complications.

In conclusion, we found that frequent high-cost users exhibit more severe CF at baseline, characterized by female sex, reduced lung function, in the peri-transplantation phase, and having liver cirrhosis with portal hypertension. This group incurs higher costs due to a higher number of hospitalizations and an increased usage of medications. Interventions targeting frequent high-cost CF users may reduce healthcare costs.

## Supplementary Information

Supplementary Information.

## Data Availability

Access to data provided by the Data Steward(s) is subject to approval, but can be requested for research projects through the Data Steward(s) or their designated service providers. Please contact https://www.popdata.bc.ca for more information about these data.
