# Supplementary material for: Factors associated with frequent high-cost individuals with cystic fibrosis and their healthcare utilization and cost patterns
Source: Sci Rep. 2023 Jun 1;13:8910. doi: 10.1038/s41598-023-35942-7 (PMC10235057; doi:10.1038/s41598-023-35942-7)
Supplement: Supplementary file 1 — Supplementary Information. [file 41598_2023_35942_MOESM1_ESM.docx]

**Supplementary Document**

**1. Additional information of probabilistic linkage process**

In probabilistic matching/linking, matching is conducted in an iterative fashion, called “passes”. The first part of a pass is called blocking (**Figure S1**, *step 1*). For blocking, we specified variables by which records from both datasets (administrative and CCFR) are compared. All records that have an exact match on these variables are considered potential matches and are separated (**Figure S1**, *step 2*). These potential matches are all on the same “block”. For example, we used date of birth as our first blocking variable. All records from both datasets which have the same date of birth were set aside. Within this block, potential matches are already similar across the blocking variable (i.e., date of birth). Now for the second part of the pass, other “linking” variables provide information that can be used to precisely tease apart true matches from false matches. For example, in our study, gender, first name and last name are possible “linking” variables. They were used to assign numeric values or weights^8^ by a statistical software to indicate the likelihood of the potential matches being a real match (**Figure S1**, *step 3*). After linking, a threshold or cut-off weight is set (e.g., 0.80) for which all records in potential pairs with weights above this threshold are considered true matches and are formally matched. For example, in **Figure S1**, Sameer Desai/Sam Desai and Jon Doe/Jon Doe will be matched because they are above the 0.80 threshold. The threshold was selected by first visually checking the distribution of weights of all potential matches and identifying a weight at which every pair above it appears to be a true match and a lower weight at which pairs are true non-matches by manual review.


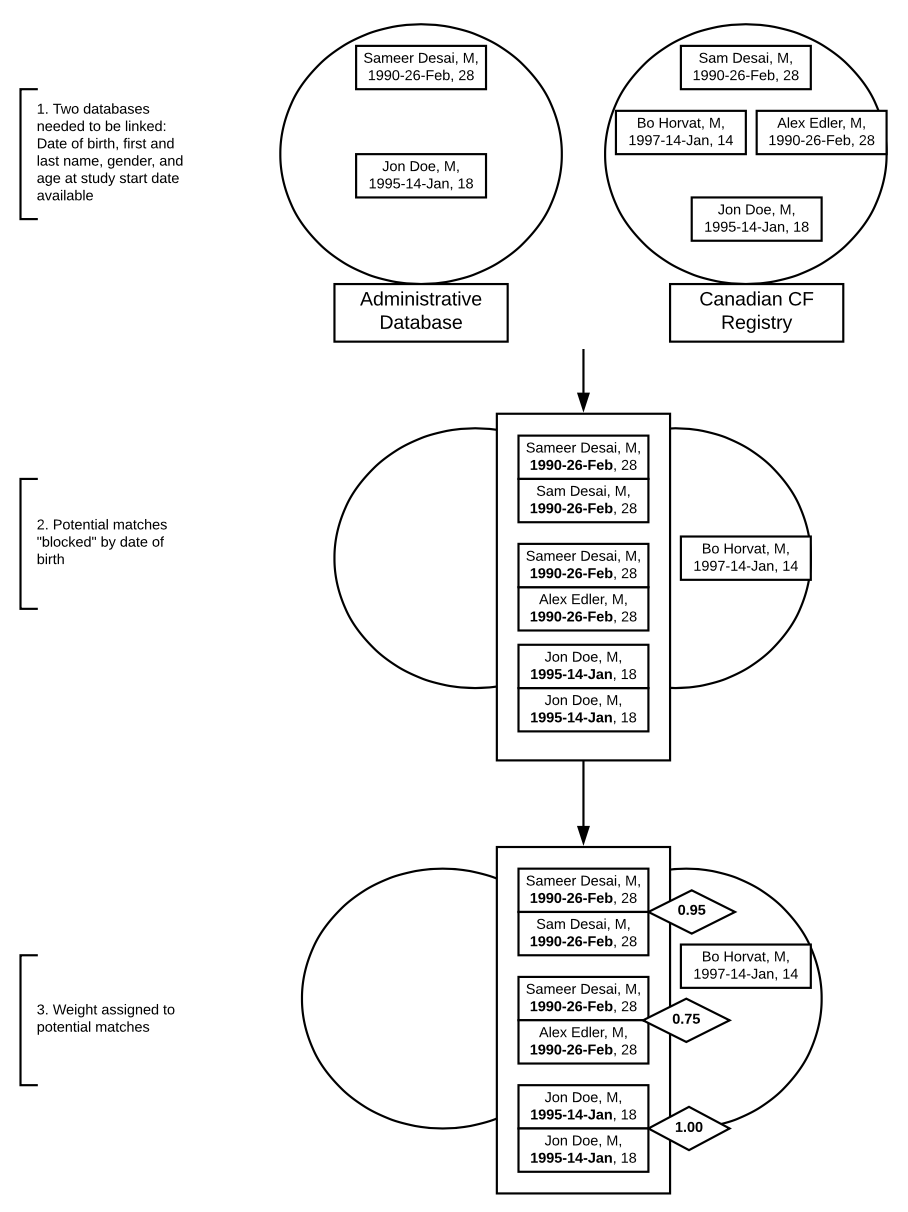


***Figure S1. First pass of probabilistic linkage using a hypothetical example***

| **Factors** | **Final Multivariable HRs**  **(95% CIs)^a^** | **HRs excluding CFTR modulator costs from total^b^** | **HRs censoring observations post-CFTR initiation** |
| --- | --- | --- | --- |
| **Female Sex (ref: Male)** | 1.97 (1.13-3.44)** | 1.57 (0.86-2.87) | 1.44 (0.74-2.77) |
| ***P. aeruginosa* (+ve)** | 1.14 (0.64-2.05) | 1.58 (0.81-3.09) | 1.61 (0.78-3.30) |
| **ppFEV_1_ category (ref: Normal)** |  |  |  |
| Mild | 0.91 (0.36-2.34) | 1.92 (0.66-5.62) | 1.49 (0.44-4.99) |
| Moderate | 2.44 (1.13-5.25)** | 3.98 (1.49-10.6)** | 4.69 (1.73-12.73)** |
| Severe | 3.71 (1.49-9.21)** | 6.95 (2.28-21.21)** | 7.85 (2.72-22.70)** |
| **Lung transplanted (ref: No)** |  |  |  |
| Peri-transplant phase | 4.23 (1.68-10.69)** | 3.61 (1.28-10.19)** | 4.52 (1.92-10.65)** |
| ≥ 2 year post-transplant | 0.87 (0.10-7.41) | 0.58 (0.12-2.83) | 1.91 (0.38-9.58) |
| **Psychiatric medication use (ref: None)** | 1.65 (0.76-3.60) | 1.84 (0.83-4.04) | 1.40 (0.56-3.49) |
| **Liver cirrhosis (ref: No)** | 10.96 (3.85-31.20)** | 10.04 (3.44-29.31)** | 4.29 (1.72-10.69)** |

***Table S1. Regression model results excluding CFTR modulator costs from total costs and censoring observations post CFTR (starting from 2013)***

***^a^ N = 59 frequent high-cost users included***

***^b^ N = 55 frequent high-cost users included***

***** = P < 0.05***

| **Factors** | **Final Multivariable HRs**  **(95% CIs)** | **Final Multivariable HRs with age included**  **(95% CIs)** |
| --- | --- | --- |
| **Female Sex (ref: Male)** | 1.97 (1.13-3.44)** | 1.98 (1.11-3.51)** |
| ***P. aeruginosa* (+ve)** | 1.14 (0.64-2.05) | 1.04 (0.57-1.90) |
| **ppFEV_1_ category (ref: Normal)** |  |  |
| Mild | 0.91 (0.36-2.34) | 1.23 (0.47-3.23) |
| Moderate | 2.44 (1.13-5.25)** | 4.12 (1.74-9.79)** |
| Severe | 3.71 (1.49-9.21)** | 7.52 (2.54-22.24)** |
| **Lung transplanted (ref: No)** |  |  |
| Peri-transplant phase | 4.23 (1.68-10.69)** | 4.72 (1.79-12.43)** |
| ≥ 2 year post-transplant | 0.87 (0.10-7.41) | 1.47 (0.16-13.51) |
| **Psychiatric medication use (ref: None)** | 1.65 (0.76-3.60) | 1.82 (0.79-4.21) |
| **Liver cirrhosis (ref: No)** | 10.96 (3.85-31.20)** | 9.88 (3.32-29.39)** |
| **Age category (ref: 6-11)** |  |  |
| 12-18 | - | 0.46 (0.18-1.18) |
| 19-40 | - | 0.37 (0.16-0.86)** |
| 40+ | - | 0.13 (0.04-0.41)** |

***Table S2. Regression model results including age category variable; clinical associations remain unchanged.***

****P< 0.25***

***** P < 0.05***

| **Factors** | **Final Multivariable HRs**  **(95% CIs)** | **HRs – frequent high-cost #1**  **(95% CIs)** | **HRs – frequent high-cost #2**  **(95% CIs)** |
| --- | --- | --- | --- |
| **Female Sex (ref: Male)** | 1.99 (1.14-3.47)** | 1.84 (0.95-3.59)* | 2.80 (1.24-6.35)** |
| ***P. aeruginosa* (+ve)** | 1.09 (0.61-1.95) | 1.25 (0.62-2.52) | 0.90 (0.39-2.06) |
| **ppFEV_1_ category (ref: Normal)** |  |  |  |
| Mild | 1.00 (0.40-2.54) | 0.96 (0.35-2.66) | 1.36 (0.41-4.52) |
| Moderate | 2.57 (1.18-5.58)** | 1.51 (0.61-3.72) | 1.20 (0.35-4.11) |
| Severe | 4.06 (1.62-10.14)** | 2.01 (0.67-6.00)* | 3.36 (0.95-11.93)* |
| **Lung transplanted (ref: No)** |  |  |  |
| Peri-transplant phase | 4.14 (1.64-10.43)** | 4.27 (1.41-12.89)** | 3.95 (1.17-13.39)** |
| ≥ 2 year post-transplant | 0.62 (0.08-5.08) | NA | NA |
| **Psychiatric medication use (ref: None)** | 1.61 (0.74-3.50) | 1.54 (0.57-4.14) | 2.51 (0.89-7.08)* |
| **Liver cirrhosis (ref: No)** | 11.00 (3.89-31.17)** | 8.28 (2.28-30.13)** | 11.02 (2.89-41.95)** |

****P< 0.25***

***** P < 0.05***

***Table S3. Primary analysis definition: 50% of follow-up time in top decile OR 2 consecutive years in top decile (N=59)***

***Frequent high-cost #1: 50% of follow-up time in top decile OR 3 consecutive years in top decile (N=40)***

***Frequent high-cost #2: 50% of follow-up time in top decile only (N=28)***

| **Factors** | **Final Multivariable HRs**  **(95% CIs)** | **6-17 years of age Multivariable HRs**  **(95% CIs)** |
| --- | --- | --- |
| **Female Sex (ref: Male)** | 1.97 (1.13-3.44)** | 1.20 (0.49-2.98) |
| ***P. aeruginosa* (+ve)** | 1.14 (0.64-2.05) | 1.68 (0.67-4.23) |
| **ppFEV_1_ category (ref: Normal)** |  |  |
| Mild | 0.91 (0.36-2.34) | 0.70 (0.19-2.59) |
| Moderate | 2.44 (1.13-5.25)** | 2.51 (0.75-8.38)* |
| **Complications (ref: No)** | - | 7.86 (2.59-21.72)** |

****P< 0.25***

***** P < 0.05***

***Table S4. Regression model results for children only. Note: Many factors (e.g., Severe lung function impairment, CFRD, transplant, psychiatric medication use were not estimable due to small numbers (<5).***

| **Factors** | **Final Multivariable HRs**  **(95% CIs)** | **>=18 years of age Only Multivariable HRs (95% CIs)** |
| --- | --- | --- |
| **Female Sex (ref: Male)** | 1.97 (1.13-3.44)** | 2.33 (1.24-4.40)** |
| ***P. aeruginosa* (+ve)** | 1.14 (0.64-2.05) | 0.88 (0.44-1.75) |
| **ppFEV_1_ category (ref: Normal)** |  |  |
| Mild | 0.91 (0.36-2.34) | 5.74 (0.70-47.5)* |
| Moderate | 2.44 (1.13-5.25)** | 10.75 (1.40-82.75)** |
| Severe | 3.71 (1.49-9.21)** | 24.34 (3.00-197.59)** |
| **Lung transplanted (ref: No)** |  |  |
| Peri-transplant phase | 4.23 (1.68-10.69)** | 2.64 (1.19-6.23)** |
| ≥ 2 year post-transplant | 0.87 (0.10-7.41) | 0.61 (0.06-6.58) |
| **Psychiatric medication use (ref: None)** | 1.65 (0.76-3.60) | - |
| **Liver cirrhosis (ref: No)** | 10.96 (3.85-31.20)** | 5.78 (2.04-16.39)** |

***Table S5. Regression model results for adults only***

****P< 0.25***

***** P < 0.05***

***
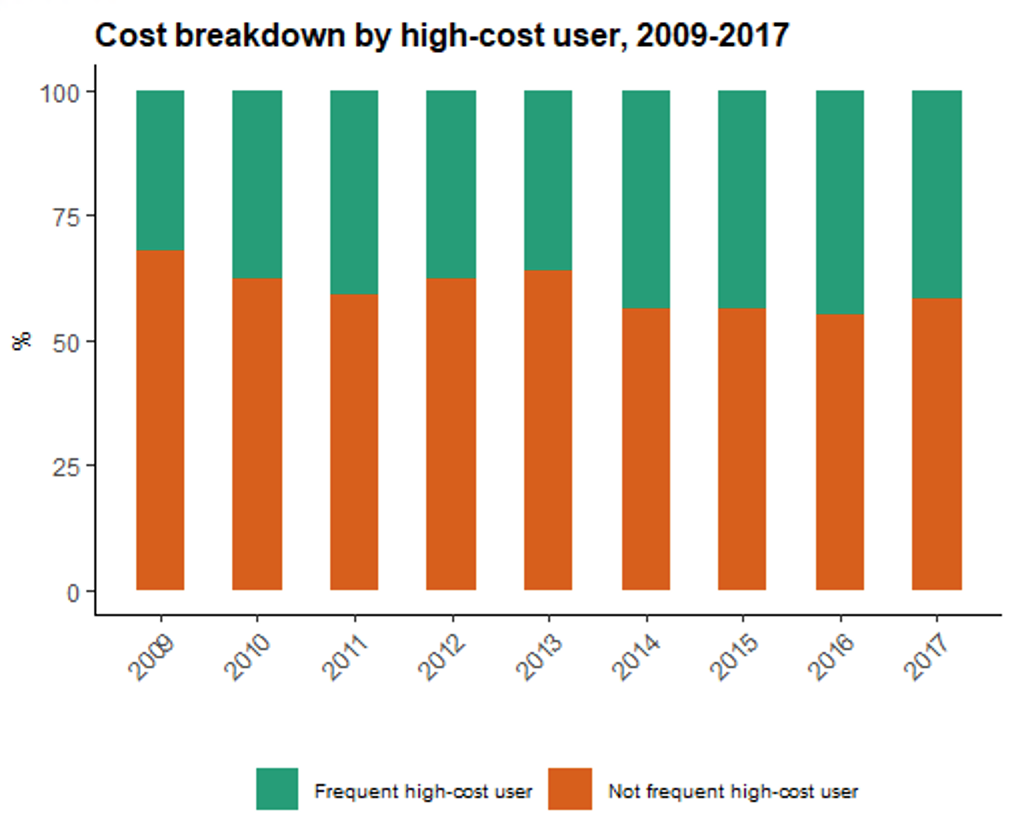
***

***Figure S2. Percentage of overall total health care costs in each study year by cost group***


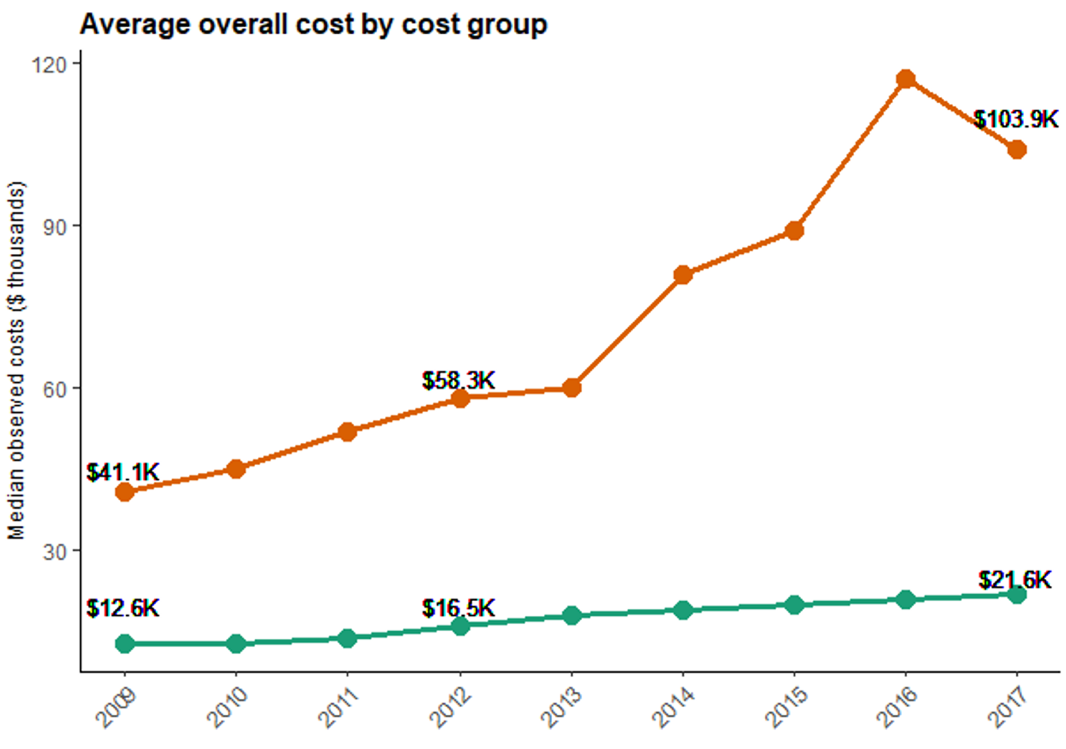


***Figure S3. Median annual (per-patient) total costs for frequent high-cost users (top line) and not frequent high-cost users (bottom line))***

***
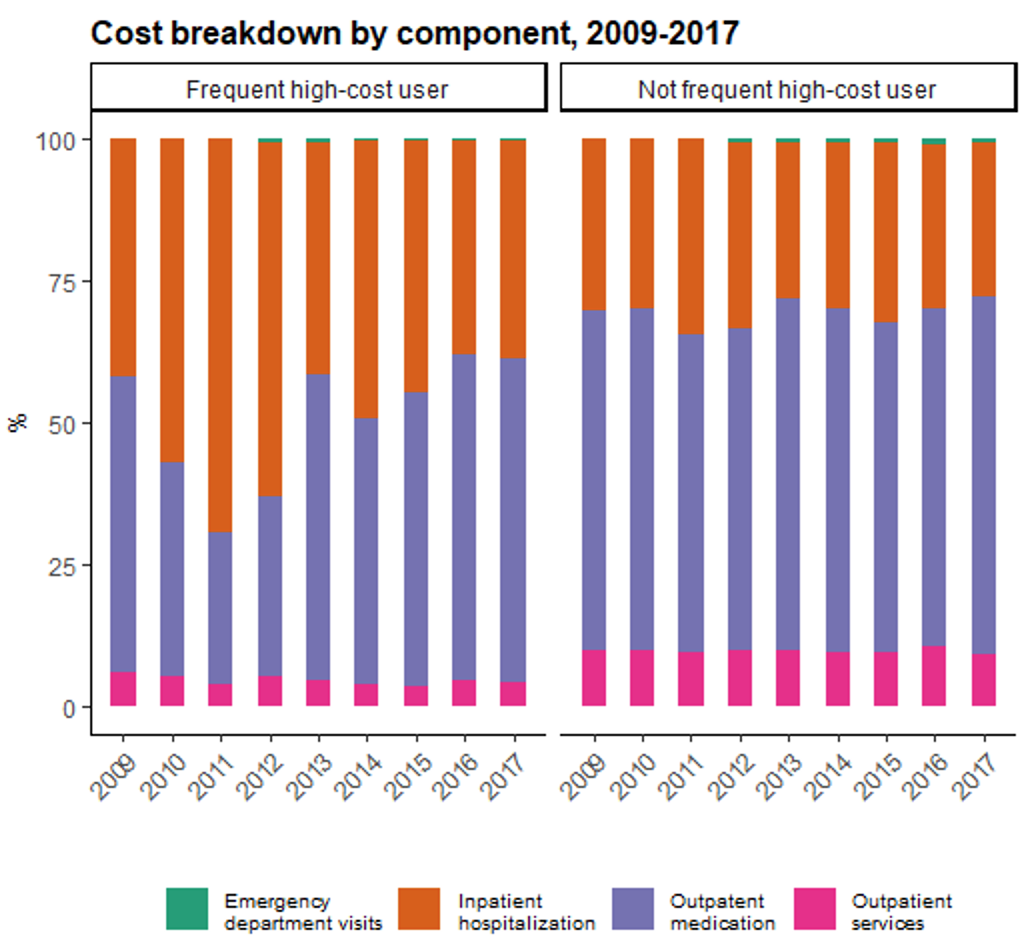
***

**Figure S4. Cost breakdown by broad service categories for each cost, 2009 to 2017.**

***
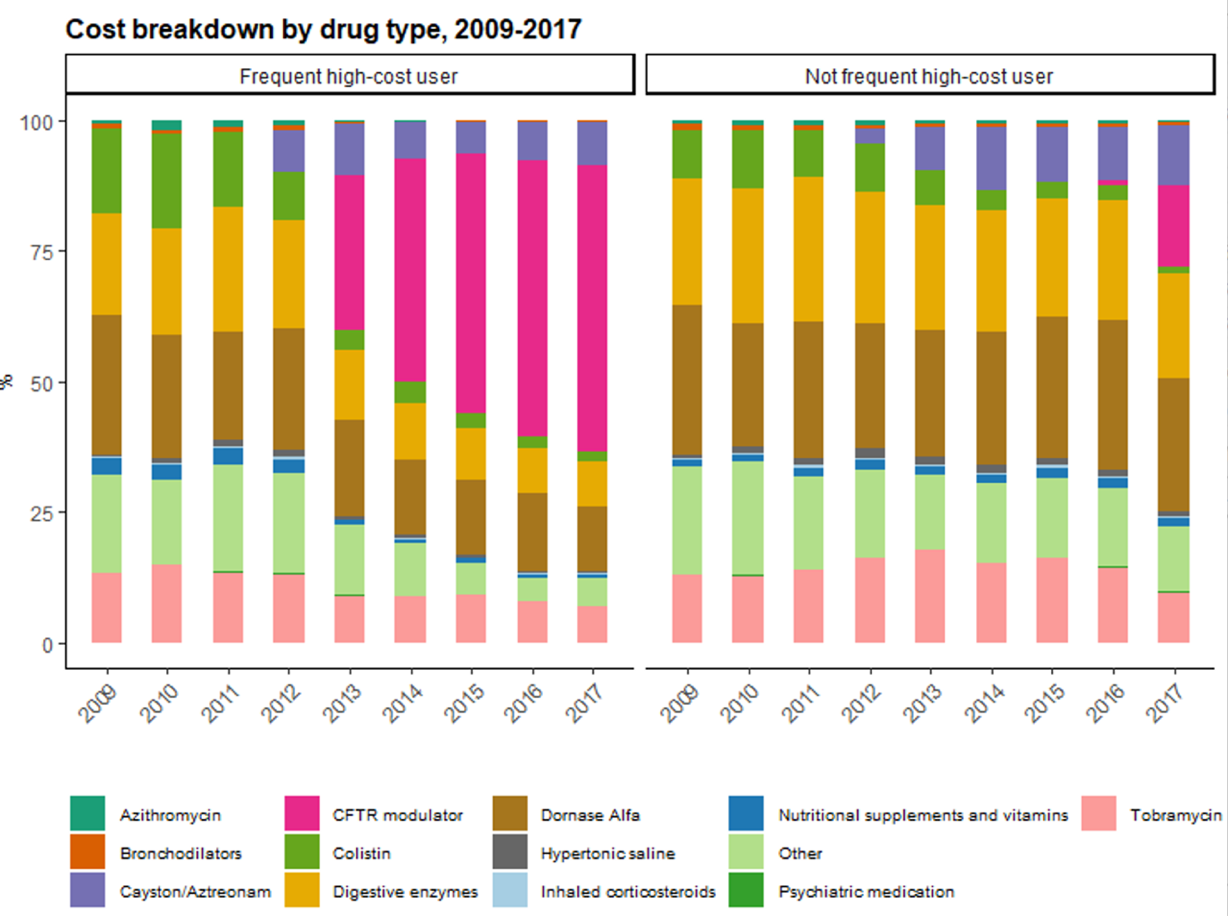
***

***Figure S5. Cost breakdown by drug type* (*n= 286 non-frequent CF cost users vs 59 frequent high-cost users*)**

***
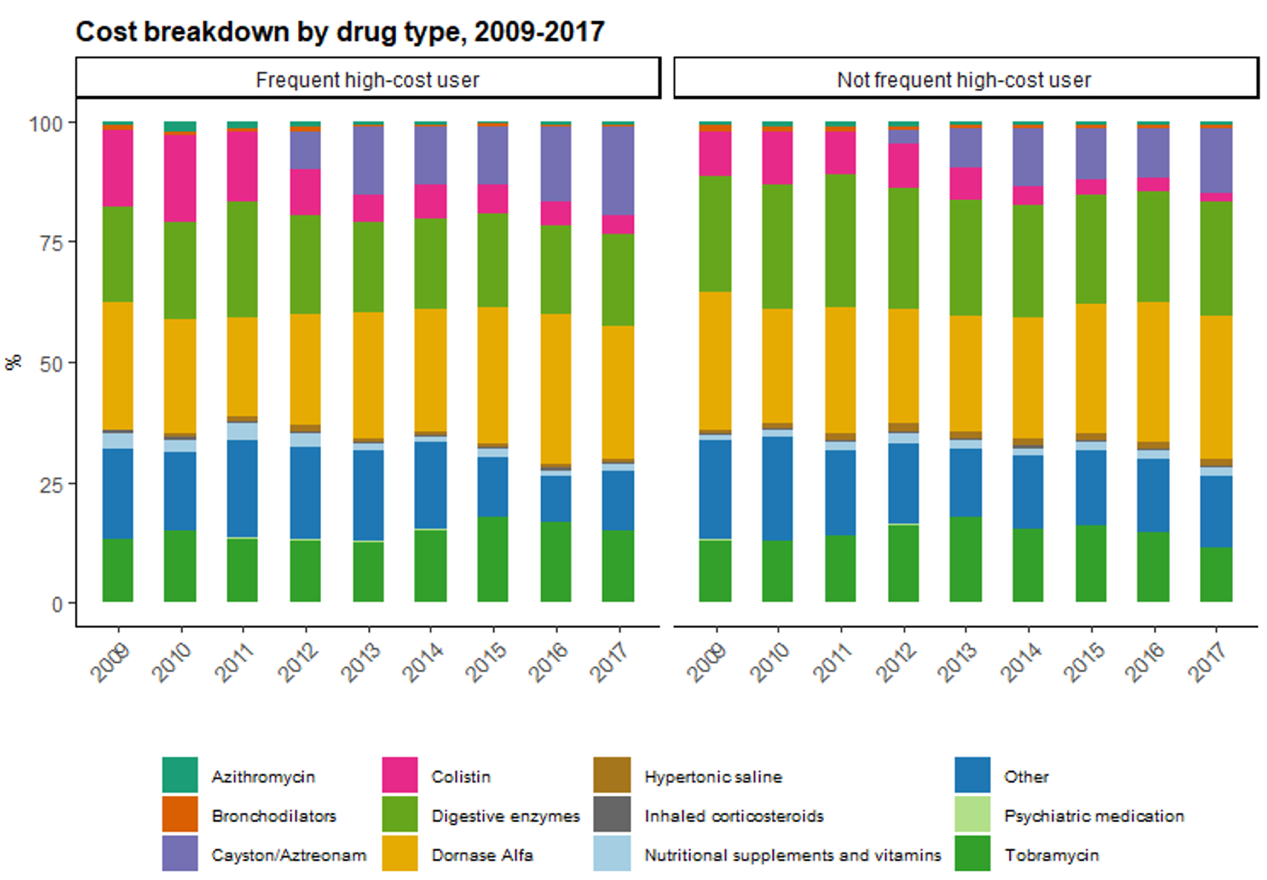
***

***Figure S6. Cost breakdown by drug type excluding CFTR modulator costs* (*n= 290 non-frequent CF cost users vs 55 frequent high-cost users*)**

**
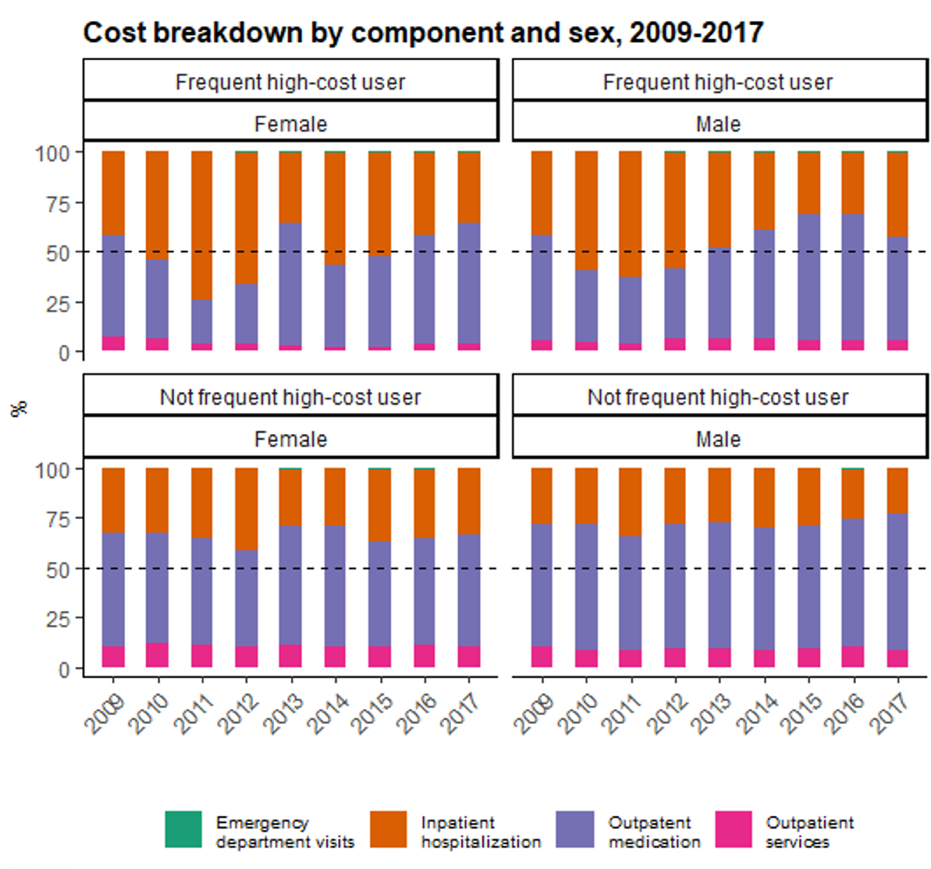
**

**
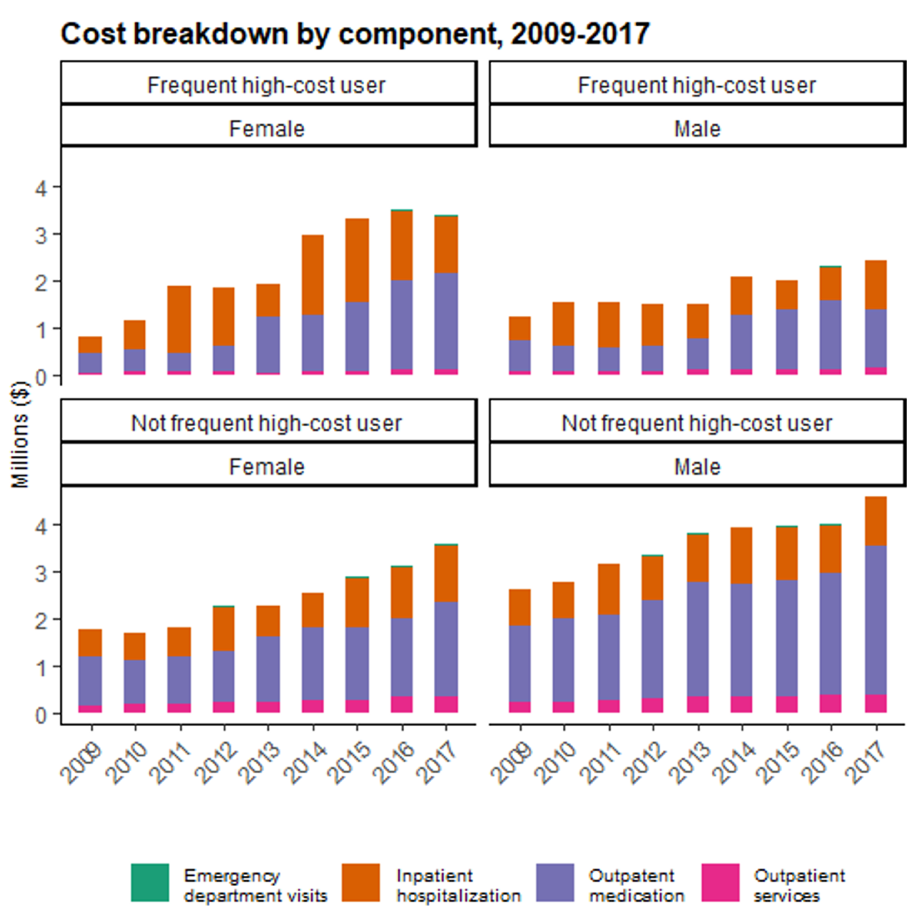
**

**Figure S7. Cost breakdown by for each cost group by sex, 2009 to 2017. Top = breakdown by percentage, Bottom = Breakdown by absolute costs**

**Exploratory analyses**

| **Characteristic** | **Not frequently high-cost (N=115)** | **Frequently**  **high-cost (N=20)** |
| --- | --- | --- |
| **Age at diagnosis,** y, median (IQR) | 0 (0-3.5) | 0.5 (0-5.5) |
| **Sex**, n (%) |  |  |
| Female | 47 (41) | 11 (55) |
| Male | 68 (59) | 9 (45) |
| **Race**, n (%) |  |  |
| Caucasian | 110 (96) | 20 (100) |
| **Primary area of residence**, n (%) |  |  |
| Fraser | 50 (43) | 7 (35) |
| Interior | 22 (19) | ≤5 (≤25) |
| Northern | ≤5 (≤4.3) | ≤5 (≤25) |
| Island | ≥ 5 (≥4.3) | ≤5 (≤25) |
| Vancouver Coastal | 22 (19) | ≤5(≤25) |
| **Presence of F508del mutation**, n (%) | 105 (91) | 17 (85) |
| **CFRD on treatment**, n (%) | c | ≤5 (≤25) |
| **Exocrine Pancreatic Insufficiency**, n (%) | 104 (90) | 18 (90) |
| ***P. aeruginosa*,** n (%) | 42 (37) | 12 (60) |
| ***B. cepacia complex,*** n (%) | ≤5 (≤4.3) | ≤5 (≤25) |
| **Underweight**, n (%) | 16 (15) | ≤5 (≤25) |
| **ppFEV_1_ category** |  |  |
| Normal | 59 (59) | 9 (53) |
| Mild | 27 (27) | ≤5 (≤25) |
| Moderate | ≥ 5 (≥8.5) | ≤5 (≤25) |
| Severe | ≤5 (≤4.3) | ≤5 (≤25) |
| **ppFEV_1_ broad category** |  |  |
| Normal | 59 (59) | 9 (53) |
| Mild-to-severe | 41 (41) | 8 (47) |
| **Liver cirrhosis,** n (%) | - | ≤5 (≤25) |
| **Nasal polyps,** n (%) | 5 (4.3) | ≤5 (≤25) |
| **No. of complications,** n (%) |  |  |
| No complications | 109 (95) | 16 (80) |
| >=1 complication | 6 (5) | ≤5 (≤20) |

***Table S6. Characteristics of pediatric individuals. No incident of DIOS, massive hemoptysis or pneumothorax reported.***

***Note: Counts 5 or less are suppressed. When counts less than 5 can be guessed with information from other categories, the smallest next category was replaced with “≥ 5 (≥%)”***

| **Characteristic** | **Not frequently high-cost (N=168)** | **Frequently**  **high-cost (N=17)** |
| --- | --- | --- |
| **Age at diagnosis,** y, median (IQR) | 1 (0-7) | 3 (0-7) |
| **Age in baseline year, n (%)** |  |  |
| Pediatrics | 86 (51) | 7 (41) |
| Adult | 82 (49) | 10 (59) |
| **Sex**, n (%) |  |  |
| Female | 71 (42) | 7 (41) |
| Male | 97 (58) | 10 (59) |
| **Race**, n (%) |  |  |
| Caucasian | 160 (95) | 17 (100) |
| **Primary area of residence**, n (%) |  |  |
| Fraser | 62 (37) | 8 (47) |
| Interior | 26 (15) | ≤5 (≤29) |
| Northern | 8 (4.8) | ≤5 (≤29) |
| Island | 29 (17) | ≤5 (≤29) |
| Vancouver Coastal | 43 (26) | ≤5 (≤29) |
| **Presence of F508del mutation**, n (%) | 151 (90) | 13 (76) |
| **CFRD on treatment**, n (%) | 23 (14) | ≤5 (≤29) |
| **Exocrine Pancreatic Insufficiency**, n (%) | 136 (81) | 15 (88) |
| ***P. aeruginosa*,** n (%) | 68 (40) | 10 (59) |
| ***B. cepacia complex,*** n (%) | 12 (7.1) | ≤5 (≤29) |
| **Underweight**, n (%) | 11 (7.2) | 3 (21) |
| **Psychiatric medication use,** n (%) | 7 (4.2) | ≤5 (≤29) |
| **DIOS,** n (%) | 11 (6.5) | ≤5 (≤29) |
| **Massive hemoptysis,** n (%) | ≤5 (≤3.0) | - |
| **Liver cirrhosis,** n (%) | ≤5 (≤3.0) | ≤5 (≤29) |
| **Pneumothorax,** n (%) | ≤5 (≤3.0) | - |
| **Nasal polyps,** n (%) | 38 (23) | 5 (29) |
| **No. of complications,** n (%) |  |  |
| No complications | 117 (70) | 10 (59) |
| >=1 complication | 51 (30) | 7 (41) |

***Table S7. Characteristics at normal-to-mild lung disease individuals only.***

|  | **2009 (N=59)** | **2010** | **2011** | **2012** | **2013** | **2014** | **2015** | **2016** | **2017**  **(N=48)** |
| --- | --- | --- | --- | --- | --- | --- | --- | --- | --- |
| **Specialist visits** |  |  |  |  |  |  |  |  |  |
| Mean no. of visits | 5.0 (5.1) | 6.3 (7.1) | 7.1(6.5) | 9.1 (7.4) | 8.5 (7.1) | 9.5 (9.6) | 9.1 (7.6) | 12.1 (11.7) | 11.9 (12.4) |
| Median no. of visits | 4 (1-6) | 4.5 (2-7) | 5.5 (3-9) | 7 (4-13) | 6 (4-11) | 7 (5-11) | 8 (5-11) | 9 (4-15.3) | 8 (3-17)* |
| **General practitioner visits** |  |  |  |  |  |  |  |  |  |
| Mean no. of visits | 7.0 (19.9) | 6.2 (16.1) | 4.3 (7.9) | 4.0 (7.4) | 4.1 (7.8) | 3.5 (5.3) | 3.0 (4.5) | 2.4 (3.7) | 2.5 (3.3) |
| Median no. of visits | 2 (1-5) | 2.5 (0-7) | 1.5 (0.3-5) | 2 (0-4) | 2 (0-4) | 1 (0-4) | 1 (1-3) | 2 (0-3) | 2 (0-3) |
| **Outpatient medication claims** |  |  |  |  |  |  |  |  |  |
| Mean no. of claims | 86 (104) | 89 (153) | 89 (156) | 92 (154) | 72 (61) | 72 (47) | 62 (44) | 79 (50) | 82 (52) |
| Median no. of claims | 61 (40-87) | 59 (30-88) | 61 (22-88) | 59 (35-89) | 58 (39-95) | 65 (40-95) | 55 (30-80) | 64 (43-103) | 62 (49-111)* |
| **Inpatient hospitalizations** |  |  |  |  |  |  |  |  |  |
| Mean no. of hospitalizations | 1.4 (1.5) | 1.9 (2.0) | 2.0 (2.5) | 2.1 (2.3) | 2.1 (2.9) | 2.0 (2.4) | 2.0 (2.4) | 1.7 (1.9) | 1.8 (1.9) |
| Median no. of hospitalizations | 1 (0-2) | 1 (0-3) | 1 (0-3) | 1 (0-3) | 1 (0-3) | 1 (0-3) | 1 (0-3.3) | 1 (0-3) | 1 (0-3)* |
| **ED visits** |  |  |  |  |  |  |  |  |  |
| Mean no. of ED visits | NA | NA | NA | 1.9 (2.8) | 1.8 (3.3) | 1.9 (3.0) | 2.0 (2.4) | 1.8 (2.6) | 1.8 (3.6) |
| Median no. of ED visits | NA | NA | NA | 1 (0-2) | 1 (0-3) | 0 (0-3) | 1 (0-3) | 0.5 (0-2.3) | 1 (0-2)* |

***Table S8. Frequent high-cost users***

|  | **2009 (n=286)** | **2010** | **2011** | **2012** | **2013** | **2014** | **2015** | **2016** | **2017 (N=261)** |
| --- | --- | --- | --- | --- | --- | --- | --- | --- | --- |
| **Specialist visits** |  |  |  |  |  |  |  |  |  |
| Mean no. of visits | 4.2 (4.6) | 3.8 (4.1) | 4.8 (4.5) | 5.7 (5 | 5.4 (4.9) | 5.4 (5.5) | 6.0 (5.2) | 6.0 (6.0) | 6.5 (7.3) |
| Median no. of visits | 3 (1-6) | 3 (1-5) | 4 (2-6) | 5 (3-7) | 4 (3-7) | 4 (2-6) | 5 (3-8) | 4 (2-8) | 5 (2-8) |
| **General practitioner visits** |  |  |  |  |  |  |  |  |  |
| Mean no. of visits | 3.4 (3.3) | 3.4 (4.6) | 3.2 (4.2) | 3.1 (3.8) | 3.3 (4.5) | 3.2 (4.2) | 2.9 (3.7) | 2.9 (3.8) | 2.9 (3.5) |
| Median no. of visits | 2 (1-5) | 2 (1-4.5) | 2 (0-5) | 2 (1-4) | 2 (0-5) | 2 (1-4) | 2 (0-4) | 2 (0-4) | 2 (1-4) |
| **Outpatient medication claims** |  |  |  |  |  |  |  |  |  |
| Mean no. of claims | 42 (40) | 42 (45) | 42 (41) | 43 (46) | 44 (40) | 43 (37) | 44 (42) | 47 (40) | 47 (47) |
| Median no. of claims | 34 (17-56) | 34 (16-59) | 37 (18-58) | 37 (18-63) | 39 (17-58) | 35 (17-57) | 37 (18-58) | 38 (20-62) | 38 (17-62) |
| **Inpatient hospitalizations** |  |  |  |  |  |  |  |  |  |
| Mean no. of hospitalizations | 0.4 (0.8) | 0.4 (0.8) | 0.5 (0.9) | 0.5 (0.8) | 0.5 (0.9) | 0.5 (1.0) | 0.5 (1.0) | 0.5 (0.9) | 0.5 (1.1) |
| Median no. of hospitalizations | 0 (0-1) | 0 (0-1) | 0 (0-1) | 0 (0-1) | 0 (0-1) | 0 (0-1) | 0 (0-1) | 0 (0-1) | 0 (0-1) |
| **ED visits** |  |  |  |  |  |  |  |  |  |
| Mean no. of ED visits | NA | NA | NA | 0.8 (1.3) | 0.9 (1.7) | 0.8 (1.9) | 0.9 (1.8) | 1.0 (1.9) | 0.8 (1.5) |
| Median no. of ED visits | NA | NA | NA | 0 (0-1) | 0 (0-1) | 0 (0-1) | 0 (0-1) | 0 (0-1) | 0 (0-1) |

***Table S9. Not frequent high-cost users***

| **Drugs** | **Claims per patient for Frequent high-cost users (N=48)** | **Claims per patient for Not Frequent**  **high-cost users**  **(N=261)** | **% Frequent high-cost users** | **% Not Frequent high-cost users** |
| --- | --- | --- | --- | --- |
| Dornase alfa | 3.6 | 2.4 | 58 | 49 |
| Cayston | 1.4 | 0.6 | 29 | 16 |
| Azithromycin | 4.6 | 2.7 | 73 | 42 |
| Tobramycin | 1.9 | 0.9 | 48 | 32 |
| Bronchodilators | 3.1 | 2.7 | 63 | 67 |
| Colistin | 0.8 | 0.2 | 21 | 7.3 |
| Hypertonic saline | 2.5 | 2.1 | 50 | 48 |
| Inhaled/oral corticosteroids | 4.8 | 2.6 | 75 | 53 |
| Digestive enzymes | 4.1 | 3.6 | 92 | 76 |
| Nutritional supplements and vitamins | 5.8 | 4.0 | 83 | 61 |
| CFTR modulators | 7.7 | 1.0 | 16 | 8.8 |
| Other | 42 | 27 | 100 | 99 |

***Table S10. Claims per patient and % utilization for different medications***

| **Year** | **No. of patients** | **Overall total costs ($ millions)** | **Overall total costs of outpatient services**  **($ millions)** | **Overall total costs of outpatient meds ($ millions)** | **Overall total costs of hospitalizations ($ millions)** | **Overall total ED visits**  **($ millions)** | **Mean total costs per patient**  **($ thousands)** |
| --- | --- | --- | --- | --- | --- | --- | --- |
| 2009 | 44 | 2.11 | 0.12 | 1.08 | 0.86 | - | 41.2 |
| 2010 | 46 | 2.75 | 0.14 | 1.02 | 1.53 | - | 45.1 |
| 2011 | 46 | 3.48 | 0.14 | 0.91 | 2.37 | - | 52.1 |
| 2012 | 48 | 3.40 | 0.18 | 1.07 | 2.09 | 0.019 | 58.3 |
| 2013 | 45 | 3.49 | 0.16 | 1.85 | 1.40 | 0.019 | 60.1 |
| 2014 | 48 | 5.13 | 0.20 | 2.35 | 2.46 | 0.021 | 81.4 |
| 2015 | 48 | 5.40 | 0.19 | 2.74 | 2.35 | 0.023 | 89.1 |
| 2016 | 48 | 5.80 | 0.26 | 3.31 | 2.17 | 0.021 | 117.4 |
| 2017 | 48 | 5.79 | 0.26 | 3.29 | 2.21 | 0.020 | 103.9 |

***Table S11. Summary statistics for frequent high-cost users by year***

| **Characteristic** | **Study sample**  **(N=345)** | **BC Sample**  **(N=500)^¶^** |
| --- | --- | --- |
| **Age at diagnosis,** y, median (IQR) | 1 (0-8) | 1 (0-5) |
| **Sex**, n (%) |  |  |
| Female | 146 (42) | 226 (45) |
| Male | 199 (58) | 274 (55) |
| **Race**, n (%) |  |  |
| Caucasian | 333 (97) | 473 (95) |
| Non-Caucasian or unknown | 12 (2.3) | 27 (5) |
| **Primary area of residence**, n (%) |  |  |
| Fraser | 126 (37) | 176 (35) |
| Interior | 60 (17) | 90 (18) |
| Northern | 18 (5.2) | 29 (5.8) |
| Island | 59 (17) | 98 (19.6) |
| Vancouver Coastal | 82 (24) | 106 (21) |
| **Presence of F508del mutation**, n (%) | 305 (88) | 439 (88) |
| **CFRD**, n (%) | 68 (20) | 141 (28) |
| **Exocrine Pancreatic Insufficiency**, n (%) | 283 (82) | 411 (82) |
| **CFTR modulator use in follow-up, n (%)** | 46 (13.3) | 54 (10.8) |
| **Transplantation status,** n (%) |  |  |
| No transplant | 322 (93) | 443 (89) |
| Transplant | 23 (7) | 57 (11) |

***Table S12. Clinical characteristics comparing current data with overall BC CF population***

**^¶^Data extracted from: Desai, S., Zhang, W., Sutherland, J. M., Zhou, X., & Quon, B. S. (2023). Economic burden of cystic fibrosis care in British Columbia. Canadian Journal of Respiratory, Critical Care, and Sleep Medicine, 1-10.**
